# Dense cold‐water coral garden of *Paragorgia johnsoni* suggests the importance of the Mid‐Atlantic Ridge for deep‐sea biodiversity

**DOI:** 10.1002/ece3.8319

**Published:** 2021-11-24

**Authors:** Telmo Morato, Carlos Dominguez‐Carrió, Christian Mohn, Oscar Ocaña Vicente, Manuela Ramos, Luís Rodrigues, Íris Sampaio, Gerald H. Taranto, Laurence Fauconnet, Inês Tojeira, Emanuel J. Gonçalves, Marina Carreiro‐Silva

**Affiliations:** ^1^ Ocean Sciences Institute ‐ Okeanos University of the Azores Horta Portugal; ^2^ IMAR Instituto do Mar University of the Azores Horta Portugal; ^3^ Department of Bioscience Aarhus University Roskilde Denmark; ^4^ Departamento de Biología Marina Fundación Museo del Mar Ceuta Spain; ^5^ University of the Azores Horta Portugal; ^6^ Portuguese Task Group for the Extension of the Continental Shelf (EMEPC) Paço de Arcos Portugal; ^7^ MARE – Marine and Environmental Sciences Centre ISPA – Instituto Universitário Lisbon Portugal; ^8^ Oceano Azul Foundation Oceanário de Lisboa Lisbon Portugal

**Keywords:** biological conservation, cold‐water corals, deep sea, Mid‐Atlantic Ridge, oceanographic processes, vulnerable marine ecosystem

## Abstract

Mid‐ocean ridges generate a myriad of physical oceanographic processes that favor the supply of food and nutrients to suspension‐ and filter‐feeding organisms, such as cold‐water corals and deep‐sea sponges. However, the pioneering work conducted along the Mid‐Atlantic Ridge failed to report the presence of large and dense living coral reefs, coral gardens, or sponge aggregations. Here, we describe the densest, near‐natural, and novel octocoral garden composed of large red and white colonies of *Paragorgia johnsoni* Gray, 1862 discovered at 545–595 m depth on the slopes of the Mid‐Atlantic Ridge, in the Azores region. This newly discovered octocoral garden is a good candidate for protection since it fits many of the FAO criteria that define what constitutes a Vulnerable Marine Ecosystem. The observations described here corroborate the existence of a close relationship between the octocoral structure and the ambient currents on ridge‐like topographies, providing new insights into the functioning of mid‐ocean ridges' ecosystems. The ubiquitous presence of biogenic and geological topographies associated with mid‐ocean ridges, which could act as climate refugia, suggests their global importance for deep‐sea biodiversity. A better understanding of the processes involved is, therefore, required. Our observations may inspire future deep‐sea research initiatives to narrow existing knowledge gaps of biophysical connections with benthic fauna at small spatial scales along mid‐ocean ridges.

## Graphical abstract

This manuscript describes the most dense, near‐natural, and novel octocoral garden composed of large red and white colonies of bubblegum corals (*Paragorgia johnsoni* Gray, 1862), discovered on the slopes of a small ridge‐like structure located on the Mid‐Atlantic Ridge, in the Azores region. The observations described here suggest a close relationship between the octocoral structure and the ambient currents on ridge‐like topographies providing new insights into the functioning of mid‐ocean ridges. The ubiquitous presence of biogenic and geological topographies associated with mid‐ocean ridges suggests their global importance for deep‐sea biodiversity and as climate refugia.

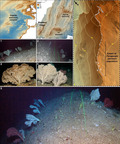

## 1 NATURAL HISTORY DISCOVERY

Mid‐ocean ridges generate a myriad of physical oceanographic processes at various temporal and spatial scales, one of which corresponds to increases in the upthrust exchange between the deep and the upper ocean (St Laurent & Thurnherr, 2007). Vertical mixing and horizontal advection favor the supply of food and nutrients to suspension‐ and filter‐feeding organisms, such as cold‐water corals and deep‐water sponges (Genin et al., 1986; Parrish & Oliver, 2020; van Haren et al., 2017). Pioneering work conducted along the Mid‐Atlantic Ridge (MAR), however, failed to observe the presence of large and dense living coral reefs, coral gardens, or sponge aggregations (Mortensen et al., 2008); likely demonstrating the lack of scientific explorations in the area.

**FIGURE 1:**
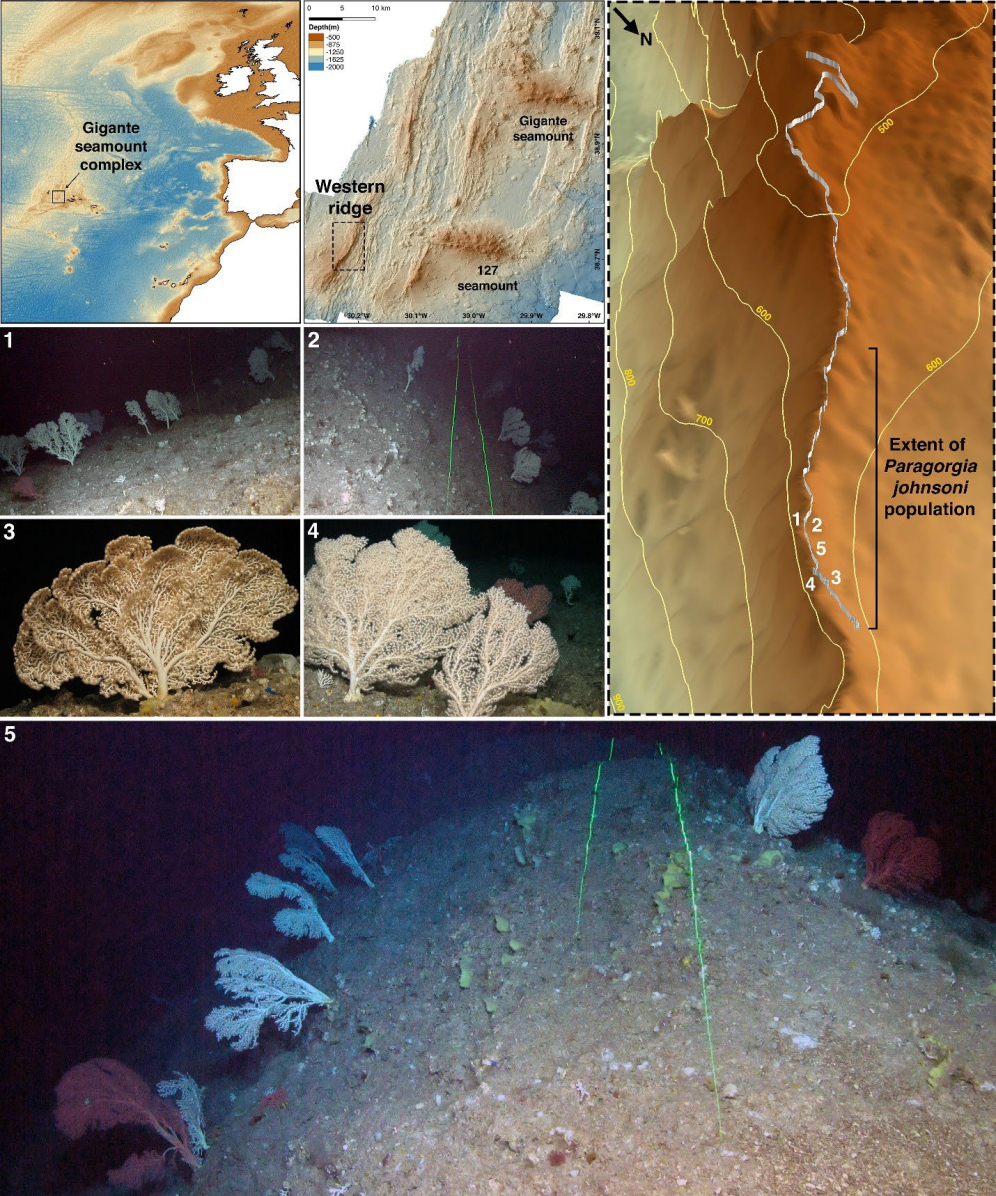
A novel octocoral garden composed of large red and white *Paragorgia johnsoni* colonies was discovered on the western ridge of the Gigante seamount complex on the Mid‐Atlantic Ridge, in the Azores region, during the Blue Azores 2018 Expedition, with the ROV *Luso,* onboard the NRP *Almirante Gago Coutinho*. The colonies were found on the slopes of both sides of the ridge, facing down the slope. The bathymetry data were collected by the Portuguese Hydrographic Institute and the Portuguese Navy. Numbers in screen captures refer to their position along the path (white line) of the ROV shown in the 3D map

Here, we describe the densest, near‐natural, and novel octocoral garden composed of large red and white colonies of *Paragorgia johnsoni* Gray, 1862 ever observed on the Mid‐Atlantic Ridge. This octocoral garden was uncovered on the slopes of a small ridge‐like structure located on the Gigante Seamount Complex in the Azores region (Figure 1) at depths of 545–595 m, during the Blue Azores 2018 Expedition onboard the NRP *Almirante Gago Coutinho* with the ROV *Luso*. Although the full extension of the octocoral garden remains unknown, it occupies a linear distance of at least 600 m along this ridge (Figure 1). The octocorals were observed growing on rocky and lithic substrates, on the slopes of both sides of the ridge, with their concave fan‐shaped structures oriented toward the deep (Figure 1), likely facing the prevailing upwelling current direction to maximize food intake (Buhl‐Mortensen & Mortensen, 2005). Observations made on the edge of the crest showed that most colonies had their backsides turned, facing opposite directions on each side of the ridge (Figure 1).

## 2 RESULTS AND PARTICULAR ASPECTS OF THE OBSERVATION

**FIGURE 2:**
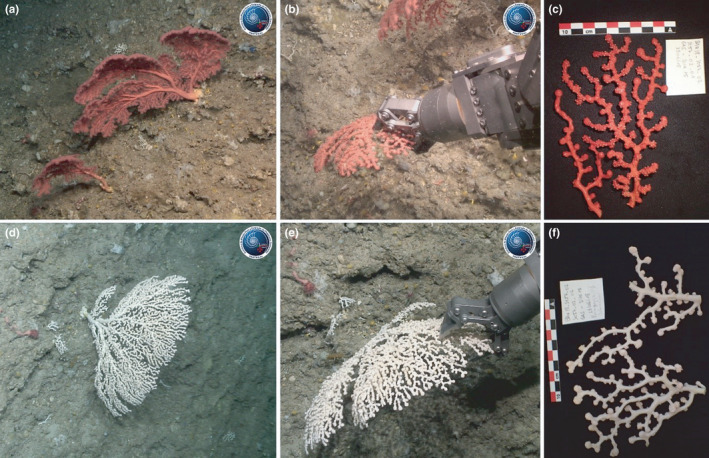
Samples of large adult *Paragorgia johnsoni* of both red (a–c) and white (d–f) variations were collected at 564 and 589 m depth with the Luso ROV hydraulic arm and manipulator claw; property of the Portuguese Task Group for the Extension of the Continental Shelf. The samples were preserved in 70% ethanol and stored at the University of the Azores' Marine Biological Reference Collection (COLETA ID 10173, 10174, and 10176)

A total of 255 colonies were reported in the video images, belonging to two clearly distinguishable morphotypes, with an approximate 4:1 ratio of white to red colonies (Dominguez‐Carrió, 2021). Samples of large adult colonies of both red (Figure 2a–c) and white (Figure 2d–f) variations were carefully collected for *ex situ* species identification. Distinct morphological characters of the colony, polyps, and sclerome were compared against original species descriptions and revision of the genus (Grasshoff, 1979a, 1979b; Sánchez, 2005). The specimens' terminal branches were smaller than 5 mm in diameter and had similar sclerome in both morphotypes. Surface cortex sclerites had a smooth ornamentation, dominated by six‐radiate sclerites averaging 0.05 mm in length. Based on these observations, both color morphotypes were identified as the octocoral species *Paragorgia johnsoni* Gray, 1862. Together with *P. arborea* (Linnaeus, 1758), these are the only two Paragorgiidae species reported for the Azores so far (Sampaio et al., 2019). Both species are commonly known as bubblegum corals because of the bulbous tips of their branches (with clumps of polyps) and are characterized by the presence of dimorphic polyps, reproductive siphonozooids, and feeding autozooids, without axial skeletal structures other than a medulla formed by unfused sclerites (Sánchez, 2005). Even though the biology, ecology, and distribution of *P. johnsoni* are far from comprehended, it appears to be widespread in the northern Atlantic Ocean, found over a wide depth (~400–4000 m depth) and temperature range (~4–13°C) (Arantes et al., 2009; Grasshoff, 1979a; Lapointe et al., 2020).

**FIGURE 3:**
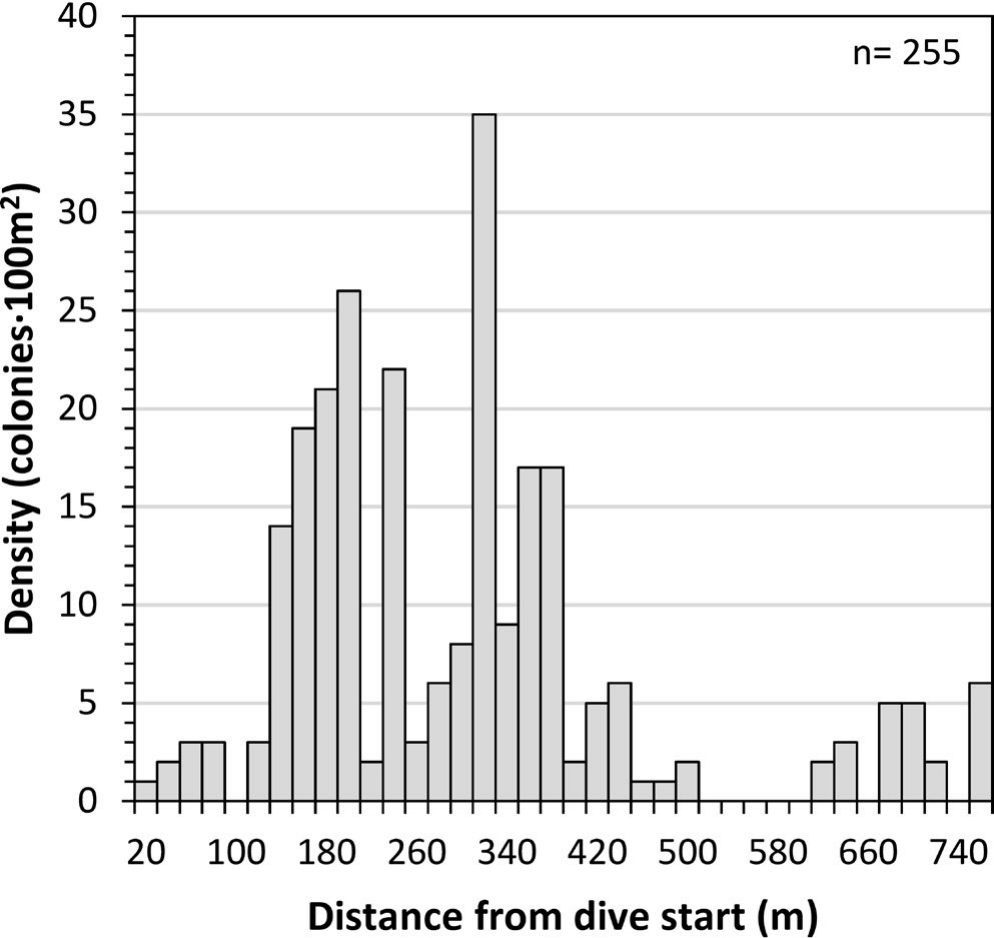
Densities of *Paragorgia johnsoni* population observed along the video transect conducted with the Luso ROV onboard of the NRP *Almirante Gago Coutinho*. Densities are shown as the number of colonies per 100 m^2^ sampling units (20 m long by 5 m width). The distance covered by the ROV was calculated using the data provided by the Ultra‐short baseline (USBL) acoustic positioning system and the average field of view obtained using the projection of the 60‐cm apart parallel lasers over the seabed

All *P. johnsoni* colonies identified along the dive were annotated and densities estimated by dividing the path of the ROV into strings of 100 m^2^ sampling units using the distance traveled over the seabed and an average field of view of 5 m. Estimated densities of *P. johnsoni* averaged 6.6 ± 8.3 col·100 m^−2^ (mean ± *SD*), with local maximum values above 30 col·100 m^−2^ (Figure 3). Although we acknowledge the caveats of this methodology, such estimates are useful to compare this coral garden with other known octocoral aggregations. In this regard, the densities reported here are of the same magnitude as those reported for other large octocoral species when forming aggregations in similar areas of the North Atlantic (e.g., *Paragorgia arborea* and *Paramuricea placomus* Linnaeus, 1758; see Buhl‐Mortensen & Buhl‐Mortensen, 2014).

**FIGURE 4:**
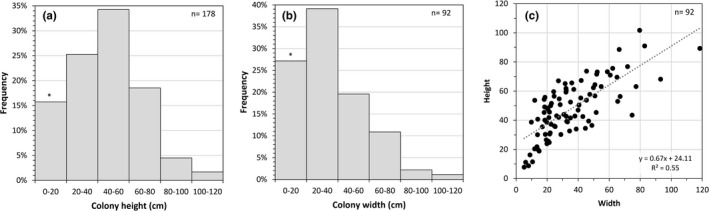
Size structure of the *Paragorgia johnsoni* population along the video transect conducted regarding (a) colony height, (b) colony width, and (c) width–height relationship. * denotes that small‐sized colonies (0–10 cm) can be underrepresented in this study

The projection of the parallel laser beams over the seabed allowed measuring the height and width of 178 and 92 colonies, respectively. Still images for each of the colonies observed were taken from the video footage and colony size was estimated using the software Macnification (Orbicule). Along the patch evaluated, *P. johnsoni* colonies measured between 6 and 107 cm in height, with an average of 45 ± 22 cm (mean ± *SD*). The most frequent sizes were between 40 and 60 cm, with at least 25% of the colonies larger than 60 cm (Figure 4a). Since small‐sized colonies of the white morph were difficult to tell apart from specimens of the white coral *Pleurocorallium johnsoni* (Gray, 1860), also detected in the same area, only colonies for which there was a high degree of confidence in their taxonomic identification were annotated and used to determine their size. This could have produced an underrepresentation of small‐sized colonies (<10 cm) in this study. The width of *P. johnsoni* ranged between 5 and 118 cm, with an average of 35 ± 21 cm (mean ± *SD*) (Figure 4b). These measurements are comparable to those reported for other large octocoral species (e.g., *Paragorgia arborea* and *Paramuricea placomus*; see Buhl‐Mortensen et al., 2010). It should be noted that although the ROV cruised very close to the seabed, the angle of the camera with respect to some of the measured colonies might have generated some underestimation of their real size. Additionally, underestimation of their natural size may have also resulted from a certain degree of structural damage. Although we acknowledge all these caveats, measurements of colony height and width are useful to understand the size structure of the population, determine the height–width relationship for this species (Figure 4c), which might be a relevant information to put the structural damage into a multispecies context, and have field data for future height–age relationships that could help infer the longevity of the population.

**FIGURE 5:**
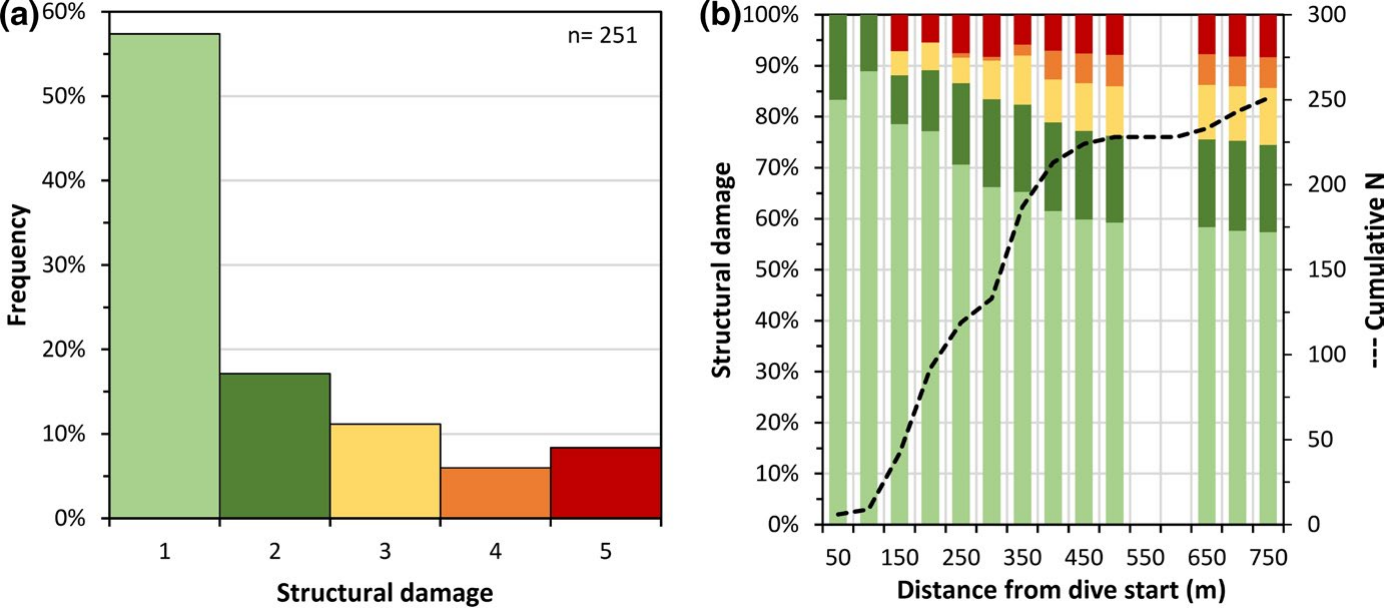
Structural damage observed on the *Paragorgia johnsoni* colonies (a) and along the video transect path (b) following Pham et al. (2014). Damages were categorized as Category 1: intact, no evidence of physical damage; Category 2: minor damage, bent and/or 1–25% physical damage (e.g., broken/missing branches); Category 3: mild damage, 26–50% physical damage; Category 4: major structural damage, 51–75% physical damage; Category 5: dead/massive structural damage, 76–100% physical damage, displaced, and/or dead. The structural damage was not assigned to four colonies because the quality of the image was not appropriate

The *P*. *johnsoni* colonies were assigned to different degrees of structural damage following an adaptation of the categories defined by Pham et al. (2014). Noteworthy, most colonies observed along the video transect were found in good condition (Figure 5a), with 75% being intact or with very minor structural damage. About 14% of the colonies were found with major or massive structural damage likely caused by physical contact with bottom longlines, the most common fishing gear in the region (Pham et al., 2014; Sampaio et al., 2012). In fact, 20 portions of lost mono‐ and multifilament longlines were observed on the video transect, some of which were in close proximity of the damaged colonies. Although the observed damage may also have been caused by other unknown natural reasons (e.g., local environmental conditions, excess turbulence, low colony fitness, or large predators), the past and present fishing footprint generally overlaps with the distribution of cold‐water corals causing severe physical disturbances (Clark et al., 2016). Even on this ridge, the observed structural damage increased along the path of the transect (Figure 5b), with more affected colonies toward shallower and more fished areas closer to the summit. However, the overall good status of this *P. johnsoni* garden suggests reduced human‐induced disturbance and a near‐pristine status of this site.

## 3 DISCUSSION AND IMPLICATIONS OF THE OBSERVATION

These observations corroborate a close relationship between octocoral structure and ambient currents on ridge‐like topographies, as *Paragorgia* colonies developed a concave fan‐shaped structure typical of tall octocorals oriented toward unidirectional current flows; in contrast to other non‐concave colonies occurring in areas with oscillating currents (Mortensen & Buhl‐Mortensen, 2005; Peccini & MacDonald, 2008). This strategy allows *Paragorgia* colonies to take advantage of increased food supplies derived from laminar, high‐velocity, and unidirectional current flows. If the described current flow versus fan shape relationship generated by either unidirectional or oscillating flows holds true, the observed shapes and orientation of colonies could serve as a proxy for predicting prevailing flows along mid‐ocean ridges. Areas of enhanced currents and food supply are particularly important for the development of benthic species in oligotrophic regions, such as the central north Atlantic Ocean.

Descriptions of *P. johnsoni* gardens like this one are rare and elusive (Lapointe et al., 2020), contrary to the related cosmopolitan octocoral species *Paragorgia arborea* (Linnaeus, 1758), for which gardens composed of dense aggregations have been reported in several locations of the North Atlantic (e.g., Sundahl et al., 2020). This discovery demonstrates that *P. johnsoni* can also form dense coral gardens, extending our knowledge of this species' ecology and revealing its potential role as a structuring species of deep‐sea ecosystems. Dense octocoral gardens are known to foster biodiversity at the local scale, providing complex three‐dimensional habitats for other deep‐sea species to thrive (Buhl‐Mortensen & Mortensen, 2005; Krieger & Wing, 2002) and, possibly, fostering the development of deep‐sea communities through ecological facilitation (Crotty et al., 2019).

The coral garden described here was found at much shallow depths (500–600 m) than many of previously reported occurrences, raising the hypothesis that shallow regions of the MAR may provide refugia from acidification impacts on *P. johnsoni* and on other similar octocoral species, as suggested for seamount summits (Tittensor et al., 2010). However, the upthrust exchange between the deep and the upper ocean may, at the same time, expose cold‐water corals to deep corrosive waters (Feely et al., 2008). The increased food supply provided by upwelling currents in ridge‐like topographies may provide the additional energy needed for calcification, allowing them to survive the projected conditions of carbonate undersaturation in deep waters (Thresher et al., 2011). These aspects of mid‐ocean ridges functioning deserve further investigation. *Paragorgia* spp. have been mostly recorded in waters supersaturated in carbonate that enable the bio‐calcification of their skeletons (Bostock et al., 2015), although several records exist in slightly carbonate undersaturated waters in the Pacific Ocean (Bostock et al., 2015; Thresher et al., 2011). The axial skeleton of Paragorgids is made of high magnesium calcite sclerites, which are particularly vulnerable to dissolution under carbonate undersaturated waters, although the octocoral tissue (coenenchyme) intertwined among unfused sclerites may provide protection from corrosive conditions (Bostock et al., 2015; Gabay et al., 2014). Not surprisingly, their suitable habitat is forecasted to be significantly reduced under future conditions of ocean acidification (Morato et al., 2020). *Paragorgia* spp. have been suggested to be long‐lived, with life spans on the scale of decades to centuries (Sherwood & Edinger, 2009), which exacerbates their vulnerability to climate and anthropogenic impacts.

This newly discovered octocoral garden is a good candidate for protection since it fits many of the FAO criteria (FAO, 2009) that define what constitutes a Vulnerable Marine Ecosystem (VME). In brief, this *P. johnsoni* garden is currently unique in the MAR (*Uniqueness criteria*), it is habitat‐forming, acting as foundation species and biodiversity refugia at a microhabitat scale (*Functional significance criteria*), it is fragile and susceptible to disruptive human activities as it is composed by large organisms with complex 3D morphologies (*Fragility criteria*), and its main structuring species possesses life‐history traits such as slow growth and long life span, which suggests a low recovery capacity (*Life‐history criteria*).

## 4 CONCLUSIONS

Our observation provides new information for understanding the functioning of mid‐ocean ridges, questioning previous observations documenting the lack of enhanced biological productivity over the MAR (Priede et al., 2013), and providing some evidence for a localized ridge effect enhancing biological productivity. This discovery may provide an important opportunity to further investigate the ecological role of large, dense, and long‐lived cold‐water corals as ecological facilitators and their capacity to signal particular nutrient and current regimes. Although our observations were obtained from some of the shallowest portions of the MAR, the lack of detailed scientific explorations in the area opens the hypothesis that these aggregations may be more common and widespread than previously expected. The ubiquitous presence of biogenic and geological topographies associated with mid‐ocean ridges suggests their global importance for deep‐sea biodiversity, conservation, and climate refugia. A better understanding of how mid‐ocean ridges can influence local biodiversity is, therefore, required.

## CONFLICT OF INTERESTS

All authors declare that they have no competing or conflicts of interest.

## AUTHOR CONTRIBUTIONS

**Telmo Morato:** Conceptualization (lead); data curation (equal); formal analysis (equal); funding acquisition (equal); investigation (equal); methodology (equal); project administration (equal); supervision (equal); visualization (equal); writing‐original draft (lead); writing‐review & editing (lead). **Carlos Dominguez‐Carrió:** Conceptualization (supporting); data curation (equal); formal analysis (equal); investigation (equal); methodology (equal); visualization (equal); writing‐original draft (supporting); writing‐review & editing (supporting). **Christian Mohn:** Formal analysis (supporting); investigation (equal); methodology (supporting); writing‐original draft (supporting); writing‐review & editing (supporting). **Oscar Ocaña Vicente:** Formal analysis (supporting); investigation (equal); methodology (supporting); writing‐original draft (supporting); writing‐review & editing (supporting). **Manuela Ramos:** Formal analysis (supporting); investigation (equal); methodology (supporting); writing‐original draft (supporting); writing‐review & editing (supporting). **Luís Rodrigues:** Data curation (supporting); formal analysis (supporting); investigation (equal); methodology (supporting); visualization (equal); writing‐original draft (supporting); writing‐review & editing (supporting). **Íris Sampaio:** Formal analysis (supporting); investigation (equal); methodology (supporting); writing‐original draft (supporting); writing‐review & editing (supporting). **Gerald H. Taranto:** Formal analysis (supporting); investigation (equal); methodology (supporting); writing‐original draft (supporting); writing‐review & editing (supporting). **Laurence Fauconnet:** Formal analysis (supporting); investigation (equal); methodology (supporting); writing‐original draft (supporting); writing‐review & editing (supporting). **Inês Tojeira:** Formal analysis (supporting); investigation (equal); methodology (supporting); writing‐original draft (supporting); writing‐review & editing (supporting). **Emanuel J. Gonçalves:** Formal analysis (supporting); funding acquisition (equal); investigation (equal); methodology (supporting); project administration (equal); writing‐original draft (supporting); writing‐review & editing (supporting). **Marina Carreiro‐Silva:** Conceptualization (supporting); formal analysis (equal); funding acquisition (equal); investigation (equal); methodology (equal); project administration (equal); supervision (equal); writing‐original draft (supporting); writing‐review & editing (supporting).

### OPEN RESEARCH BADGES

This article has earned an Open Data, for making publicly available the digitally‐shareable data necessary to reproduce the reported results. The data is available at www.doi.org/10.5281/zenodo.4727163.

## Data Availability

Data used in this work are archived in https://zenodo.org/deposit/4727164.
